# CAR T cell therapy for central nervous system solid tumors: current progress and future directions

**DOI:** 10.3389/fimmu.2025.1600403

**Published:** 2025-08-15

**Authors:** Yaroslav Kaminskiy, Vitaly Degtyarev, Alexey Stepanov, Michael Maschan

**Affiliations:** ^1^ Department of Oncology and Pathology, Karolinska Institutet, SciLifeLab, Solna, Sweden; ^2^ Dmitry Rogachev National Medical Center of Pediatric Hematology, Oncology, and Immunology, Moscow, Russia; ^3^ Shemyakin-Ovchinnikov Institute of Bioorganic Chemistry, Russian Academy of Sciences, Moscow, Russia

**Keywords:** CNS tumors, glioma, DMG, DIPG, glioblastoma, CAR T, CAR T solid tumors, brain cancer

## Abstract

Central nervous system (CNS) tumors are the second most common type of cancer in children and remain the leading cause of mortality in pediatric oncology. For patients with high-risk CNS tumors, standard treatments often prove ineffective, with survival rates being less than 10%. Hence, there is an urgent need to develop alternative treatment strategies for this patient population. Globally, numerous clinical trials are actively investigating a range of novel therapeutic approaches, from pharmacological and immunological therapies to physical modalities targeting the tumor. Among these emerging therapies, CAR T cell therapy has shown great promise, with the first objective clinical responses already reported. This review aims to evaluate the current landscape of CAR T cell therapy for pediatric CNS tumors, focusing on clinical efficacy, toxicity profiles of systemic and locoregional delivery, antigen heterogeneity, and key challenges in clinical implementation. We provide a comprehensive analysis of reported clinical trials, including not only CAR T cell studies but also investigations involving tumor-infiltrating lymphocytes (TILs), NK and lymphokine-activated killer (LAK) cells, offering a broader perspective on immunotherapeutic approaches for CNS malignancies.

## Introduction

CAR T cells are autologous or allogeneic lymphocytes engineered to express a chimeric receptor that targets a specific antigen on the surface of tumor cells. Upon binding of the CAR T cell to the tumor antigen, it becomes activated leading to cytotoxicity, cytokine secretion and proliferation (1). The scope of CAR T cell therapy has significantly expanded, particularly in the treatment of hematologic malignancies. In parallel, numerous clinical trials are ongoing to assess the efficacy and safety of CAR T therapy in solid tumors. However, several challenges remain, including the immunosuppressive tumor microenvironment, CAR T cell trafficking limitations, tumor antigen heterogeneity, antigen loss, T-cell exhaustion and treatment-related toxicity (2–4).

Additional challenges arise from the blood-brain barrier (BBB), which restricts the delivery of many therapeutic agents from the bloodstream into the CNS (5). The CNS is now recognized as an immunologically dynamic system, with the BBB and microglia forming the first line of immune defense. Peripheral immune cells, such as lymphocytes and monocytes, are typically absent in the CNS under normal conditions but can infiltrate through the BBB during pathological processes and be detected in the cerebrospinal fluid (CSF) (6). Indeed, systemically administered CAR T cells have also been detected in the CSF, indicating their capacity to cross the BBB (7). The toxic effects of systemic CAR T cell administration, such as cytokine release syndrome (CRS) and neurotoxicity, are well-documented, yet are less understood in the context of locoregional delivery into the CNS (8).

**Figure 1:**
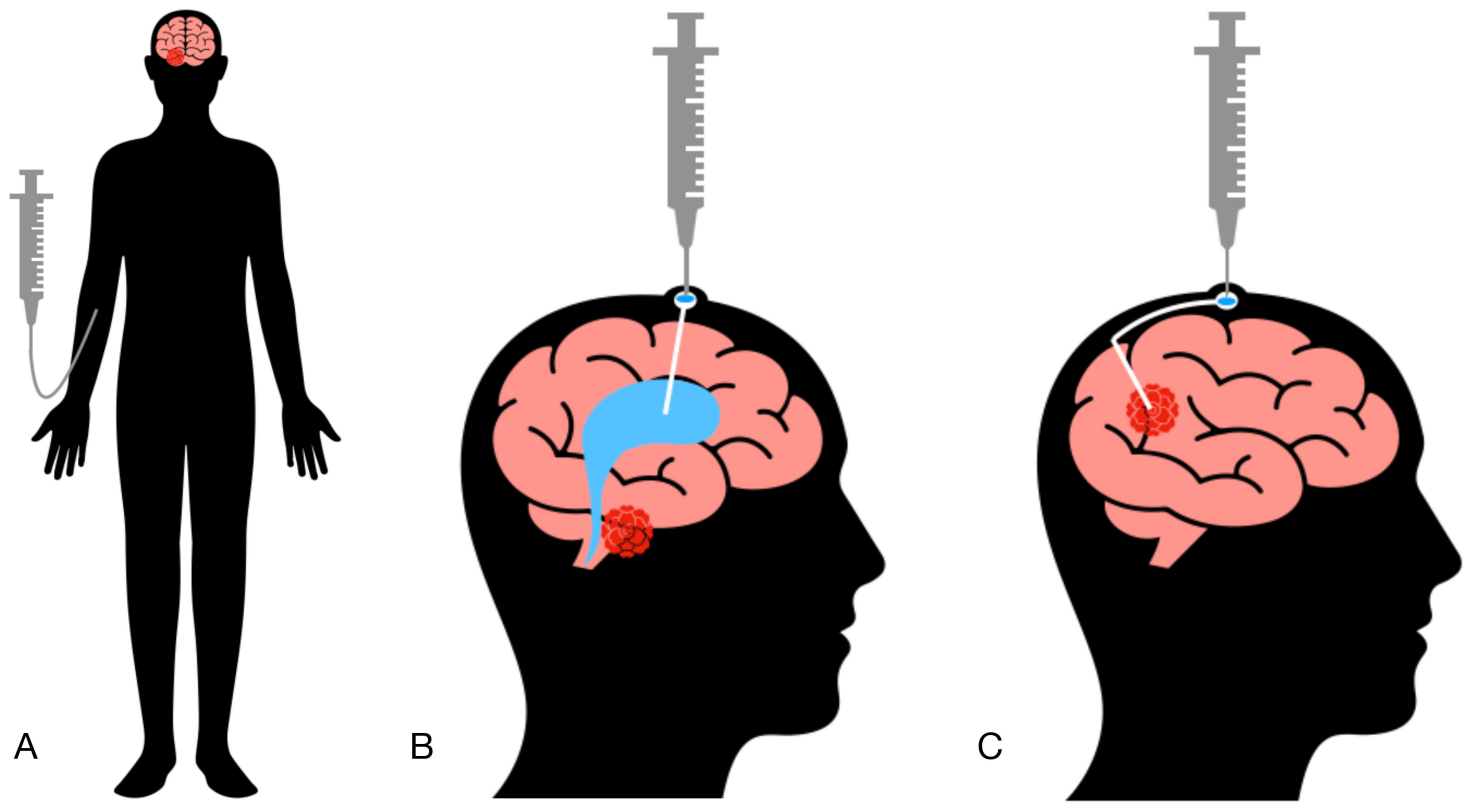
Methods of cellular product administration. **(A)** Intravenous administration (IV). **(B)** Intraventricular administration (ICV). **(C)** Intratumoral administration (IT).

In neuro-oncology, implantable systems are frequently utilized for drug delivery, allowing chemotherapeutic agents to be administered directly into the brain ventricles or the tumor site (9). Consequently, CAR T cells can be infused intravenously, intraventricularly, or locally into the postoperative tumor cavity (**Figure 1**, **Table 1**). Local drug administration enables bypassing the blood-brain barrier but is associated with certain risks. According to published data, the incidence of non-infectious complications with this delivery method reaches 33%, while infectious complications occur in 27% of cases (10). The most common non-infectious complications include intracranial hemorrhage, malfunction of the implanted catheter, and neurotoxic effects of administered drugs (11). Infectious complications are predominantly bacterial in origin, though cases of fungal and viral catheter-associated encephalitis have also been reported (12). Thus, strict adherence to catheter insertion and maintenance protocols is critical to minimizing the risk of these complications (13).

**Table 1:** Cellular therapy clinical trials in CNS tumors.

Publication	Antigen	Cell product	Conditions	Treatment	Injection route	Injection details	Additional therapy	Antitumor activity	Interesting observations
Brown et al. (15)	IL13Rα2	IL13Rα2-BBζ CAR T cells	rHGG (41 GBM, 6 AC, 2 DMG, 1 DAC, 7 grade 3 glioma)	2e6-2e8 cells (3–4 weekly doses)	IT + ICV	Rickham reservoirs (cells + 5 min saline flush)		2/58 CR; 2/58 PR; 29/58 SD	75% transduction efficiency; CAR T cells in blood; CD3+ T cells correlated with enhanced survival after therapy; ICV beneficial for multifocal tumors and IT beneficial for unifocal tumors; IFN-γ, CXCL9 and CXCL10 increase in CNS; Best OS in dual IT/ICV delivery and CD62+ Tn/mem enrichment trial arm
Bagley et al. (16); Bagley et al. (17)	IL13Rα2+EGFR	EGFR-IL13Rα2-BBζ bicistronic CAR T cells	18 multifocal rGBM (EGFR+)	1-2.5e7 cells (up to 2 doses)	ICV	Ommaya reservoir		8/13 tumor shrinkage; 1PR, 8SD	Pseudoprogression in 2 patients; CAR T cell expansion (less after second dose); CAR T cells in blood; grade 3 toxicity in 56% patients; manageable acute neurotoxicity 12-48h post-infusion (different from ICANS and TIAN); 2.5e7 cells max tolerated dose; IFN-γ, IL-2,IL-6, and TNF-α in CSF
Brown et al. (18)	IL13Rα2	IL13-zetakine+ CD8+ CTLs (GR KO, allogeneic)	6 rGBM (IL13Rα2+)	1e8 cells (4 doses over 2 weeks)	IT	Rickham reservoir (0.5 ml cells over 10 min + 1 ml PFNS flush over 2h)	IL-2 + dexamethasone	4/6 tumor necrosis	no antigen loss; potential allo-rejection
Brown et al. (19)	IL13Rα2	IL13Rα2-BBζ CAR T cells	1 multifocal rGBM (70% IL13Rα2+ tumor cells)	0.2-1e7 cells (6 IT + 10 ICV doses every 1–3 weeks)	IT+ICV	Rickham reservoir		1/1 CR (regression of 7 tumors)	IT injection prevented local but not distant tumor recurrence; predominantly CD4+ CAR T cells; no CAR T cells in blood; antigen loss leading to relapse
Brown et al. (20)	IL13Rα2	IL13-zetakine+ CD8+ CTLs	3 unifocal rGBM	1e8 cells (2 x 2-week cycles of 6 IT doses)	IT	Rickham reservoir (2 ml cells over 5–10 min + saline flush); externalized catheter (2 ml cells over 4h)		3/3 with antitumor signs (necrosis, antigen loss, no recurrence at resection border)	antigen loss
Gust et al. (21)	EGFRvIII + EGFR	EGFR-BBζ CAR T cells	3 rHGG, 1 AT/RT (over 10% EGFR+)	1-2.5e7 cells (5–10 weekly doses)	IT or ICV	Ommaya reservoir (5 ml cells over 5 min + saline flush)	–	1/4 SD	over 60% transduction efficiency; CAR T cells in blood but not in CSF, no CAR T cell infusion in over 50% of enrolled patients due to rapid disease progression
Choi et al. (22)	EGFRvIII + EGFR	EGFRvIII-BBζ CAR T cells (anti-EGFRwt TEAM secretion)	3 rGBM (EGFRvIII+ IDHwt)	1e7 cells (1–2 doses)	ICV	Ommaya reservoir (10 ml cells over 10 min)	–	3/3 tumor shrinkage	antigen loss; CAR T cells in blood
Bagley et al. (23)	EGFRvIII	EGFRvIII-BBζ CAR T cells	7 GBM (EGFRvIII+)	2e8 cells (up to 3 doses every 3 weeks)	IV	–	pembrolizumab	6/7 antigen decrease	21% transduction efficiency; no CAR T cell expansion; intratumor CAR T cells in one patient with earliest surgery (post 7 days) after infusion
Durgin et al. (24)	EGFRvIII	EGFRvIII-BBζ CAR T cells	1 rGBM (60% EGFRvIII+)	9.2e7 cells	IV	–	–	1/1 antigen decrease	antigen decrease; CAR T persistence for 29 months; CD3 tumor infiltration post therapy
Goff et al. (25)	EGFRvIII	EGFRvIII-28BBζ CAR T cells	18 rGBM (EGFRvIII+)	1e7-6e10 cells (up to 10 doses)	IV	–	cyclophosphamide + fludarabine + IL-2	1/18 long term survival	66% transduction efficiency; severe hypoxia at highest dose (without IL-2) likely due to congestion of pulmonary vasculature
O’Rourke et al. (26)	EGFRvIII	EGFRvIII-BBζ CAR T cells	10 rGBM (6-90% EGFRvIII+)	1-5e8 cells	IV	–	–	1/10 SD; 2/5 antigen loss; 5/7 antigen decrease	20% transduction efficiency; antigen loss or decrease (potentially attributable to chemotherapy); 9/10 SD after 28 days; CAR T cell expansion; CAR T tumor infiltration; IDO1, PD-L1, IL- 10 upregulation post therapy
Burger et al. (27)	HER2	HER2-28ζ CAR NK92 cells (irradiated)	9 rGBM (HER2+)	0.1-1e8 cells	IT	2 ml cells injected into resection cavity wall by 50–100 ul	–	5/9 SD	no antigen loss; PD all in lower dose group; 7 weeks PFS; 31 weeks OS
Vitanza et al. (28)	HER2	HER2-BBζ virus-specific T cells	1 AA and 2 ependymoma	1-2.5e7 cells (3 x 3-week cycles of 3 doses)	IT or ICV	CNS catheter	–	1/3 SD; 3/3 local CNS inflammation	CCL2 and CXCL10 increase in CSF and blood
Ahmed et al. (29)	HER2	HER2-28ζ virus-specific T cells	17 rGBM (HER2+)	1.7e6 - 1.7e8 cells (up to 6 doses every 6–12 weeks)	IV	–	–	1/16 PR; 7/16 SD; 5/16 pseudoprogression	39% transduction efficiency; no CAR T cell expansion but 1 year persistence
Vitanza et al. (30); Vitanza et al. (31)	B7-H3	B7-H3-BBζ CAR T cells (DHFR mutein)	21 DIPG (H3K27M+)	0.1-1e8 cells (10–81 doses every 2 weeks)	ICV	Ommaya reservoir	–	1/18 PR; 15/18 SD; 8.6 months OS improvement	CAR T cells in CSF but not blood; CAR T cell enrichment due to methotrexate selection during manufacturing; CCL2, CXCL10, GM-CSF, IFN-γ, IL-15, IL-1α, IL-6, and TNFα upregulation in CSF
Tang et al. (32)	B7-H3	B7-H3-BBζ CAR T cells	1 rGBM (heterogeneous B7-H3 expression)	0.4-2e7 cells (6 weekly doses)	IT	Ommaya reservoir (1 ml cells over 10 min + 1 ml PFNS flush over 5 min)	–	1/1 tumor shrinkage	probable antigen loss; limited expansion in later infusion cycles
Tang et al. (33)	B7-H3	B7-H3-BBζ CAR T cells	1 anaplastic meningioma	0.2-1.5e7 cells (3 weekly doses)	IT	Ommaya reservoir (1 ml cells over 10 min + 1 ml PFNS flush over 5 min)	–	1/1 tumor stabilization, necrosis and antigen decrease at infusion site	no trafficking to distant metastases; no CAR T cells in blood
Gu et al. (34)	GD2 + PSMA	GD2-28BBζ CAR T cells (iCASP9); PSMA-28BBζ CAR T cells (iCASP9)	5 rGBM, 1 rDMG	0.6-3.5e8 cells	IV	–	cyclophosphamide + fludarabine	4/6 tumor shrinkage; 3 CR; 3 SD	CAR T cell expansion but no correlation with response; CRS in 3 patients; no ICANS; low tumor burden in CR
Lin et al. (35)	GD2	GD2-BBζ CAR T cells (with or without C7R)	8 DMG, 2 medulloblastoma, 1 AT/RT	0.6-5e7 cells (up to 4 doses every 6 weeks)	IV	–	cyclophosphamide + fludarabine + anakinra	2/11 PR; 5/11 SD; 9/10 improved neurologic deficit (3 weeks after infusion)	3/5 SD tumor necrosis; all patients without C7R progressed after 1st infusion; longer PFS in C7R group; CAR T cell expansion and persistence in group with and without C7R; intratumor C7R transgene and residual GD2 detected in one patient analyzed; grade 4 CRS in one patient
Monje et al. (36); Ramakrishna et al. (37); Majzner et al. (8);	GD2	GD2-BBζ CAR T cells (iCssp9)	10 DIPG and 3 spinal DMG (H3K27M+)	1-5e7 cells (IV + 0–11 ICV monthly doses)	IV + ICV	Ommaya reservoir	cyclophosphamide + fludarabine (for IV only)	1/11 CR; 7/11 tumor shrinkage; 9/11 clinical improvement; 6.4 months OS improvement	57% transduction efficiency; no antigen loss; *in vivo* CAR T cell expansion; CAR T tumor infiltration in PD case after IV dose; regression of spinal cord tumor but not metastasis after IV dose; Tregs and MDSCs increase post therapy; dose limiting toxicity for IV but not ICV; manageable TIAN in all patients after ICV infusion correlated with CCL2 upregulation; PD associated with TGF-β upregulation
Liu et al. (38)	GD2	GD2-28BBζ CAR T cells (iCasp9)	8 rGBM (GD2+)	0.3–2e8 cells IV and 2.6-6.4e6 cells IT	IT + IV	Rickham reservoir	cyclophosphamide + fludarabine	4/8 PR; 1/8 SD; 1/8 pseudoprogression with antigen decrease	36% transduction efficiency; 7/8 patients with unmethylated MGMT; PD in (more advanced)? patients with IT infusion; CAR T cell expansion; T cell and macrophage infiltration after infusion
Qin et al. (39)	EphA2	EphA2-BBζ CAR T cells	3 rGBM (EphA2+)	6e7 cells	IV	–	cyclophosphamide + fludarabine	1/3 SD; 1/3 early tumor shrinkage	pulmonary edema likely due to lung EphA2 expression; CAR T cell expansion
Litten et al. (40)	MMP2	CLTX-28ζ CAR T cells	rGBM and AA (MMP2+)	0.8-1.5e8 cells (3 weekly doses)	IT + ICV	?		?	chlorotoxin (CLTX)-directed CAR T cells
Zhai et al. (41)	CD44 + CD133	CD44-CD133 CAR T cells (IL7R; 4th generation)	4 rGBM	0.1-1e8 cells (up to 8 weekly doses)	IT?	Ommaya reservoir		tumor shrinkage	
Yao et al. (42)	–	TILs (anti-PD1 antibody secretion)	21 rGBM	monthly doses?	IV	–	?	1/7 PR and 2/7 SD in PD1-TIL group; 3/14 SD in TIL group	16.1 vs 11.2 months OS PD1-TIL against TIL; significantly improved OS in clinical responders (30.9 vs 10.7 months)
Quattrocchi et al. (43)	–	TILs	3 GBM, 3 (all recurrent)	0.3-1e9 cells (2 doses with 2 weeks in between)	IT	Ommaya reservoir (3–5 ml cells in saline)	IL-2	3/6 PR; 2/6 SD	
Kitahara et al. (44)	–	CTLs (autologous)	4 GBM, 1 AOD	5e7 cells (up to 13 doses twice per week)	IT	Ommaya reservoir (2 ml cells in HBSS)		2/5 PR and 50% tumor shrinkage	
Kruse et al. (45)	–	CD8+ T cells (alloreactive)	2 GBM, 2 AOD, 1 AA (all recurrent)	0.3-7.5e8 cells (up to 5 x 2-week cycles of 2–3 doses)	IT or ICV	Rickham reservoir (2–8 ml cells in HBSS with IL-2)	IL-2	3/5 SD	both GBM patients progressed and died (one from local and another from distant recurrence)
Ishikawa et al. (46)	–	NK cells	3 GBM, 4 AA, 1 AOD, 1 AOA (all recurrent)	0.2–3.7e9 cells IV and 0.4-2.3e9 cells IT (up to 3 doses over 9 months)	IT + IV		IL-2 + IFNb	3/9 PR 4/9 SD after 1 dose; 4/9 tumor shrinkage	
Lim et al. (47)	*-*	CIK cells	14 rGBM	2-6e9 cells (24 doses every 2 weeks)	IV	–	–	4/14 SD; 1/14 initial radiographic improvement	10 months PFS; 22.5 months OS; 5/14 patients survived over 2 years
Kong et al. (48)	–	CIK cells	91 GBM	0.1-2e10 cells (4 doses once a week, 4 doses every 2 weeks, 6 doses every 4 weeks)	IV	–	radiotherapy + temozolomide	2.7 months PFS improvement	8.1 vs 5.4 months PFS against chemotherapy alone
Dillman et al. (49)	–	LAK cells	33 GBM	1e9 cells	IT	clot placement	IL-2	OS improvement (20.5 months with a 1-year survival rate of 75%)	20.5 vs 15 months OS against standard of therapy; superior survival for patients received higher numbers of CD3+/CD16+/CD56+ cells
Dillman et al. (50)	–	LAK cells	40 r GBM	1-3e9 cells	IT	Ommaya reservoir or clot placement	IL-2	3.9 months OS improvement	17.5 vs 13.6 months OS against standard of therapy
Hayes et al. (51)	–	LAK cells	28 GBM or AA	0.5-5e9 cells (2 monthly doses)	IT	Ommaya reservoir (4 ml over 10 min)	IL-2	6.2 months OS improvement in grade 4 gliomas	eosinophilia correlated with survival
Sankhla et al. (52)	–	LAK cells	10 GBM or AA (all recurrent)	1-2.5e6 cells (up to 5 doses every 5 days)	IT or lumbar subarachnoid injection (for multifocal)		IL-2	2/10 PR	cystic transformation in responded patients
Hayes et al. (53)	–	LAK cells	12 GBM, 4 AA, 3 GS (all recurrent)	0.5-1e9 cells (up to 2 x 6-week cycles of 3 doses)	IT	5 ml cells	IL-2	2/19 CR; 4/19 PR	IL-2-related CNS toxicity at 2.4 MIU dose; eosinophils infiltration
Blancher et al. (54)	–	LAK cells	5 rGBM	3 doses every other day after surgery	IT	–	IL-2	no apparent benefit	IL-2 related toxicity
Jeffes et al. (55)	–	LAK cells	19 rGBM	2-58e9 cells (2 doses during surgery)	IT	–	IL-2 + phytohemagglutin	3/11 tumor shrinkage;	
Lillehei et al. (56)	–	LAK and ASL cells	9 GBM, 2 AA (all recurrent)	0.5-9e9 cells (two monthly doses)	IT	Rickham reservoir or clot placement (10–20 ml)	IL-2	no apparent benefit (10/11 died of progression)	9 months OS; local tumor growth and lack of lymphocytes after infusion in 3 evaluable patients;
Ingram et al. (57); Ingram et al. (58)	–	LAK cells	83 rMG	1e8-2e9 cells	IT	15–20 ml cells in plasma	76/83 initial response?; tumor shrinkage in several patients;	25/83 were living; 18/83 no evidence of recurrence;	
Merchant et al. (59)	–	LAK cells	13 rGBM	0.5-5e9 cells (2 doses with 1–2 weeks in between)	IT	Ommaya reservoir or multiple injections into surrounding tissue (3–5 ml cells in saline)	IL-2	5/13 SD, necrosis, lymphoid infiltration	
Jacobs et al. (60)	–	LAK cells	6 MG	5e7-1e10 cells	IT	№20 blunt brain cannula (5–15 ml cells in HBSS with multiple injections into surrounding tissue)		no apparent benefit	

In the following sections, we summarized cellular therapy clinical trials in CNS tumors and try to identify common trends on several key aspects, including: initial antitumor response, availability of clinically validated antigens, post-infusion antigen escape, delivery route, toxicity profile, dosing, and the characteristics of the tumor microenvironment before and after treatment.

## Cellular therapy clinical trials in CNS tumors

CNS tumors represent the most frequently targeted solid tumors in recent and ongoing cellular therapy trials (14). **Table 1** provides a comprehensive summary of published clinical trials investigating cellular therapies for CNS tumors, with the majority focusing on high-grade gliomas (HGGs), particularly primary and recurrent glioblastoma (pGBM/rGBM), diffuse intrinsic pontine glioma (DIPG), and diffuse midline glioma (DMG). Here, we analyzed a total of 42 reported clinical trials, including 23 using CAR T/NK cells, 2 using tumor-infiltrating lymphocytes (TILs), 2 using unmodified CD8+ T cells, 1 using unmodified NK cells, and the remainder involving lymphokine-activated killer (LAK) cells.

These studies employed various approaches regarding the delivery method of the cellular product, the use of lymphodepleting chemotherapy, and different CAR T cell dosing strategies. The primary focus of all trials was to evaluate the safety and tolerability of the therapy, while the secondary objective was often to assess its efficacy.

## Initial antitumor response

Until recently, adoptive cellular therapy has demonstrated limited clinical benefit in CNS tumors when assessed by progression-free and overall survival (PFS/OS). One of the most promising trials showed that only 4 out of 58 patients with recurrent HGG responded, with additional 29 out of 58 experiencing stable disease following a course of CAR T cell therapy (15). However, relatively limited clinical benefit may be misleading when assessing the full potential of adoptive cell therapy in CNS tumors because adoptively transferred cells do exhibit the capacity to shrink tumor lesions, even if this is not yet enough to generate a robust clinical response. In fact, most CAR T and TIL trials involving local delivery—whether intrathecal (IT) or intraventricular (ICV)—have demonstrated at least transient radiographic improvement or tumor shrinkage in more than 50% of patients, with the exception of those reported by Gust et al. (21) and Vitanza et al. (28, 30). Indeed, in the most recent H3K27M+ DMG trial, significant tumor shrinkage was observed in 7 out of 11 patients with clinical improvement observed in 9 out of 11 patients (36). In general, tumor shrinkage was observed across different trials independent of target antigen and therapeutic dose. These findings suggest that even at low doses, CAR T cells show discernible antitumor activity. Unfortunately, this activity is almost always transient, with patients ultimately progressing and succumbing to the disease. The key challenges in CNS tumors are likely poor CAR T cell persistence and rapid antigen escape, both of which are well-known mechanisms of tumor evasion from CAR T cell therapy (61, 62). In the case of CNS tumors, antigen escape is probably secondary to poor persistence, as it tends to occur more frequently in patients who have received multiple therapeutic doses.

Overall, while current antitumor responses in CNS tumors have been limited, the ability of CAR T cells to cause tumor shrinkage—even temporarily— is promising and highlights the importance of continued research into improving cell persistence and reducing antigen escape to achieve better clinical outcomes.

## Several clinically validated antigens

**Figure 2:**
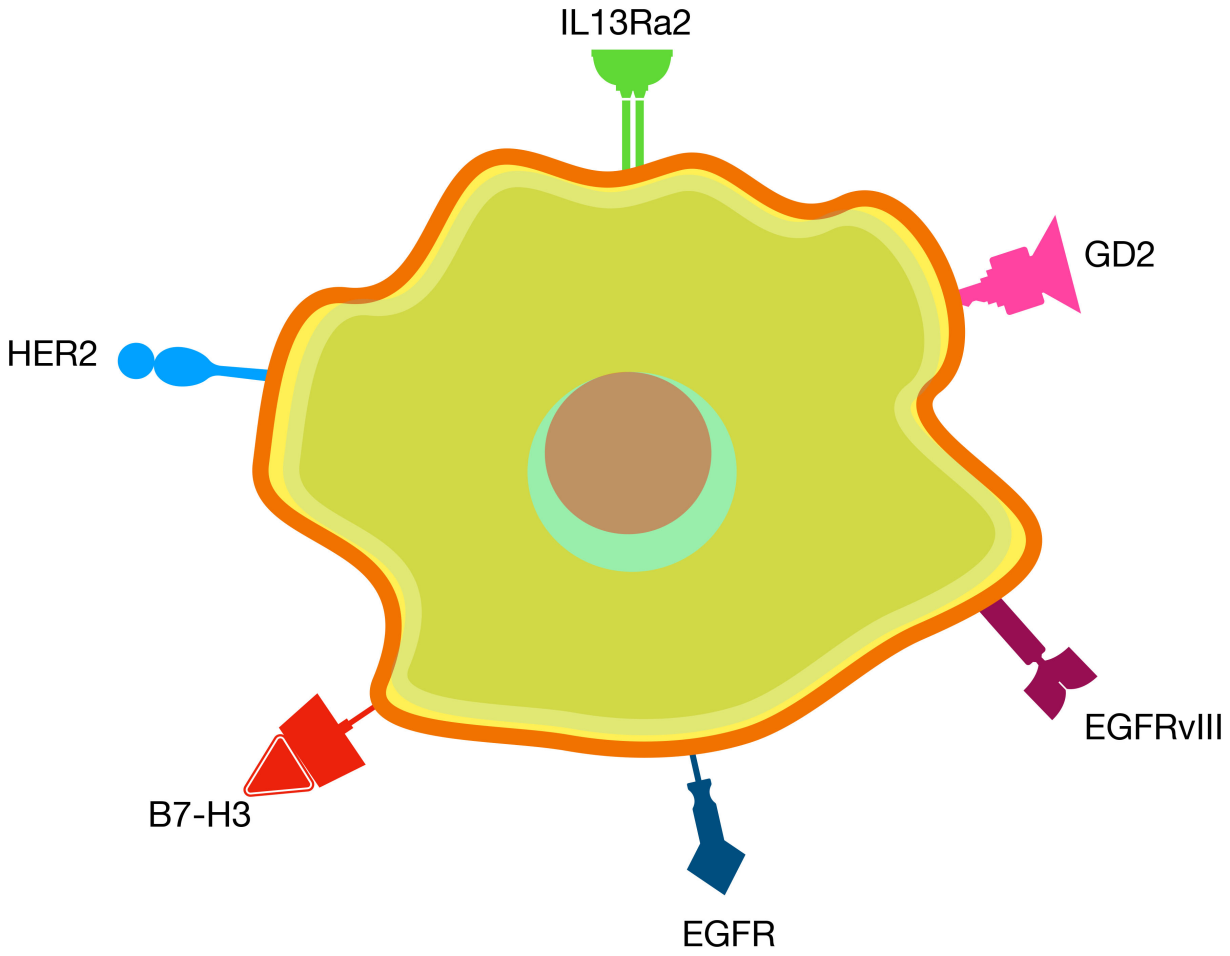
Clinically validated targets for CAR T therapy in CNS tumors.

Unlike many other solid tumors where tumor-specific antigens are scarce, gliomas have 6 clinically validated CAR T antigens that, when targeted, demonstrated tumor regression and were found to be safe when administered locally (**Table 2**) These targets include IL13Rα2 (63–69), EGFRvIII (70–72), EGFR (71, 73–77), HER2 (64, 74, 78–81), B7-H3 (82–87), and GD2 (86, 88–91) (**Figure 2**, **Table 2**).

**Table 2:** Expression frequency of clinically validated targets for CAR T therapy in CNS tumors.

Antigen	Glioblastoma	DMG/DIPG	Medulloblastoma	Ependymoma
IL13Rα2	60-78%	70-84%	67%	67-86%
EGFRvIII	34-63%	17-55%	–	–
EGFR	40-60%	27-67%	42-79%	30-60%
HER2	42-80%	60-90%	40-70%	92%
B7-H3	55-76%	88-100%	80%	70%
GD2	58-85%	80%	82%	–

IL13Rα2 is a transmembrane protein subunit of the interleukin-13 receptor, playing a crucial role in immune response regulation by binding to IL-13 (92). It is significantly overexpressed in tumor cells, with minimal expression in normal tissues. EGFR and its variant EGFRvIII belong to the epidermal growth factor receptor family, promoting signaling pathways that stimulate cell proliferation (93). EGFRvIII is a tumor-specific mutation of EGFR, resulting from an in-frame deletion of exons 2 to 7. EGFRvIII is commonly expressed in gliomas and other tumors but is absent in normal tissues, whereas EGFR is found in normal tissues but exhibits elevated expression levels in cancers (94). HER2, another member of the EGFR family, is overexpressed in various tumors, including breast cancer, and has limited expression in normal tissues (95). B7-H3 is an immune checkpoint molecule with immunosuppressive properties. While its expression in normal tissues is limited, B7-H3 is highly overexpressed in many adult and pediatric tumors, making it a promising target for immunotherapy (96). GD2, a disialoganglioside from the glycosphingolipid family, enhances tumor cell adhesion, invasiveness, and immunosuppression (97). While primarily expressed in the central and peripheral nervous systems under normal conditions, GD2 is overexpressed in numerous malignant diseases across both adults and children (98).

Although there is a recognized degree of antigenic heterogeneity in gliomas, with only a subset of tumor cells expressing any given antigen, at least one of the antigens from the list is expected to be expressed in most tumors (71). For instance, Barish et al. estimated that 93% of high grade glioma cases expressed at least one of IL13Rα2, HER2 or EGFR antigens (72% at least two) (99).This makes multitargeting as a clear path forward for enhancing therapeutic efficacy, with approaches targeting multiple pathways simultaneously already being evaluated in both clinical and preclinical trials (26, 92, 100–104).

EGFRvIII, EGFR, and HER2 are more frequently expressed in adult gliomas, while IL13Rα2, B7-H3, and GD2 appear to be suitable candidates for both adult and pediatric CNS tumors. In pediatric CNS tumors, B7-H3 and GD2 are the most frequently observed antigens, followed by IL13Rα2, with HER2 and EphA2 being less common (105). GD2 is expressed in approximately 80% of DIPG and medulloblastoma cases, making it a promising target for these tumors (88, 89). EphA2 is expressed in 60 - 90% of GBM cases and so far EphA2-CAR T cells have been tested with IV administration at relatively low dose (10e7 cells) (39, 106, 107). Although the treatment was considered safe, there were reports of pulmonary edema, likely caused by EphA2 expression in lung tissue. Local administration of EphA2 CAR T cells might solve this problem by limiting systemic migration.

New CAR T targets for glioma currently tested in preclinical and clinical studies include: CD44 (41), CD70 (108), CD97 (109), CD133 (41), CD147 (110), C.TNC (DIPG) (111), CSPG4 (GBM) (112), GPC2 (DIPG, medulloblastoma) (113), EphA3 (114), MMP2 (40, 115), PD-L1 (GBM) (116), PTGFRN (117), ROBO1 (GBM) (71, 118, 119).

A recent study demonstrated that a peptide derived from the most common mutation DMGs, H3.3K27M (found in 70% of cases), can be effectively targeted by T cells in the HLA-A*02:01 context (120). Combined with evidence that most high-grade gliomas express HLA class I complexes, this supports the potential of adoptive T cell therapy as a promising strategy for targeting gliomas with the H3.3K27M mutation (105). Other, less conventional targets include CMV antigens such as pp65 or IE1-72 (expressed in 67% of adult HGG), ligands for NK receptor NKG2D (71, 107, 121) and BTN2A1/BTN3A ligands for γ9δ2 T-cell receptor (122).

Altogether, the availability of several validated antigens for gliomas provides an excellent opportunity to address antigen escape through multitargeting CAR T strategies; however, the impact of tumor heterogeneity on treatment durability remains a critical unanswered question. Future studies should investigate how initial antigen expression levels and spatial differences across metastatic sites influence long-term responses, potentially through paired biopsy analyses.

## Post-infusion antigen escape

Antigen escape is a well-recognized mechanism that contributes to CAR T therapy failure (61). Several studies have documented antigen loss following CAR T cell infusion in CNS tumors, including IL13Rα2, EGFRvIII, EGFR, and B7-H3 (19, 20, 22, 23, 32). It is worth noting that concurrent chemotherapy may also contribute to decreased antigen expression (at least for EGFR) and, therefore, decreased antigen levels in trials combining CAR T cells with chemotherapy might be misleading (26). Given the availability of several clinically validated antigens, antigen escape can be mitigated through the use of bi- or tri-specific CARs, or other multitarget CAR designs (22, 100, 123). Although there is a possibility that all validated antigens could eventually be lost, to date no study has demonstrated such an occurrence, making this a promising therapeutic strategy.

In summary, implementing multitarget CAR T cell approaches offers a feasible solution to combat antigen escape in CNS tumors. The promising preclinical and clinical findings to date suggest that combining multiple antigen targets could significantly enhance the therapeutic potential of CAR T cell therapy.

## Benefits of local delivery

Evidence suggests that local (IT or ICV) delivery outperforms IV administration in terms of both antitumor response and toxicity profile. With a few exceptions, IV-infused cells have shown limited CNS and tumor infiltration, as well as reduced antitumor activity (**Table 1**). Additionally, systemic administration restricts the range of suitable target antigens and therapeutic doses. For example, EphA2—a promising glioma target—as well as HER2 are expressed in lung epithelium, posing a risk of off-target toxicity similar to that observed in early HER2 CAR T trials (39, 124). Lowering the therapeutic dose may reduce toxicity risks but could also compromise antitumor efficacy (29). Local delivery of HER2 CAR T cells, by contrast, was well tolerated and shows signs of antitumor activity (28, 125). Furthermore, local delivery does not require lymphodepletion, which is often a prerequisite for IV administration. The superiority of local delivery has also been demonstrated in multiple preclinical brain tumor models (123, 126–128).

It appears that ICV administration is more effective for multifocal disease, as IT administration resulted in very limited distal infiltration of CAR T cells (15, 16, 19). On the other hand, ICV is also suitable for both unifocal and multifocal diseases, especially given that up to 35% of glioblastomas become multifocal (129). Although there has been no head-to-head clinical comparison between IT and ICV delivery, several trials have demonstrated this even at low therapeutic doses (8, 15, 16, 19, 22, 36, 37). It appears that a combination of IT and ICV infusions could be advantageous in combating both local and distal recurrence, as dual administration is both feasible and safe, with early indications suggesting a clinical benefit (15).

Rickham and Ommaya reservoirs are widely used devices for local delivery of therapeutic agents into the cerebrospinal fluid (CSF) or brain tissue (130). Both have shown comparable efficacy in administering adoptive cell therapy in CNS tumors (**Table 1**). As a general practice, between 0.5 to 8 ml of cell products were delivered through these devices (IT or ICV) over about 10 minutes, followed by a flush with saline to ensure complete delivery. In certain cases, cell products were injected directly into the surrounding brain tissue near a resected tumor cavity.

Overall, the evidence strongly supports local (IT and/or ICV) delivery as an effective method for enhancing CAR T cell therapy efficacy in CNS tumors. Implementing these strategies could obviate the need for improving CAR T cell migration and brain infiltration by delivering the cells close to the tumor site. This not only achieves higher local concentrations of CAR T cells in the tumor microenvironment but also reduces off-target effects ultimately leading to better patient outcomes.

## Favorable toxicity profile

Local (IT, ICV) infusions have shown excellent tolerability in most studies, with very few instances of grade 3 or higher adverse events (**Table 1**). With the exception of Bagley et al. (17), neither recent CAR T trials nor older LAK studies exhibited evident dose-limiting toxicity, even at the highest doses (10e10 cells per infusion). Typically, CAR T cell doses were in the range of 10e7 to 10e8 cells per infusion, which is lower compared to LAK infusions that ranged from 10e9 to 10e10 cells. Additionally, the proportion of CAR+ T cells in infusion products rarely reached 100%, suggesting that the effective dose might be even lower. These observations imply the potential for dose escalation to 10e10 CAR T cells or more per injection. Although direct comparison between CAR T cells and LAK cells dosing may not be appropriate—considering the differences in mechanisms of action—LAK cells were often injected with high doses of IL-2 and still showed a favorable safety profile, indicating that further dose escalation for CAR T cells could be possible. Of course, CAR T cell manufacturing capabilities must be considered, as expanding sufficient cells for multiple doses at 10e10 CAR T cells could be challenging. Interestingly, early studies with LAK cells also demonstrated that local delivery of high IL-2 doses is better tolerated than IV delivery. In summary, the favorable toxicity profile of local CAR T cell infusions provides an opportunity for dose escalation to enhance antitumor efficacy while maintaining safety. Further exploration of optimized dosing regimens could maximize therapeutic benefit.

## Multiple dosing

CAR T cell expansion, commonly observed 1–3 weeks post-infusion and associated with antitumor responses in hematologic malignancies, was noted in several CNS trials but did not clearly correlate with clinical benefit (16, 26, 32, 36–38). Similarly, long-term persistence of CAR T cells (up to 29 months) was observed in two studies, but this was achieved with IV administration and without any obvious clinical response (24, 29). Hostile tumor microenvironment and limited tumor trafficking are likely barriers to achieving sustained CAR T cell expansion and persistence in CNS tumors. To overcome this, it is now recognized that multiple-dose infusion regimens are needed to achieve meaningful clinical responses. Recent CAR T trials have administered up to 18 doses, with weekly infusions being the most common strategy.

Implementing multiple dosing strategies appears essential for achieving clinical responses in CNS tumors. Given the challenges with CAR T cell persistence and expansion in the tumor microenvironment, frequent and repeated dosing could increase the likelihood of achieving durable tumor control and maximizing the therapeutic potential of adoptive cellular therapies.

## Post-treatment tumor microenvironment

Following CAR T cell infusion, several soluble factors were observed to be upregulated in CSF, including IFN-γ (8, 15, 30, 31, 38), IL-6 (8, 21, 30, 38), CCL2 (28, 30), CXCL10 (15, 28, 30, 31), TGFβ (8, 26, 36), GM-CSF (31) and TARC (31). IL-6 and TGFβ are known to inhibit T cell immunity, and CCL2 can facilitate the recruitment of myeloid cells and Tregs potentially contributing to acquired resistance against CAR T therapy (131–133). Indeed, myeloid cell infiltration post-infusion was reported in two studies, while IDO1, PD-L1, and IL-10 upregulation in the TME was documented in one study, further supporting the emergence of compensatory inhibitory pathways post-CAR T therapy (26, 38).

Hence, post-infusion TME inhibitory mechanisms, such as upregulation of inhibitory cytokines and increased myeloid cell infiltration, emphasize the need to engineer CAR T cells resistant to these challenges.

## Novel strategies for CAR T cell engineering

In general, tumor microenvironment of high-grade gliomas is considered immunologically ‘cold’ due to its limited effector T cell infiltration (134). These tumors are characterized by a significant presence of suppressive myeloid cells and Tregs, as well as harsh conditions such as hypoxia, nutrient deprivation, and the accumulation of immunosuppressive factors like extracellular adenosine, galectin-1, FGF, IDO, IL-6, IL-10, PD-L1, PGE2, and TGFβ (64, 71, 103, 107, 115).

Several strategies have been proposed to counteract the inhibitory effects of the tumor microenvironment and enhance CAR T cell survival and persistence in glioma. These include constitutively active IL-7 receptor (C7R) (35, 135), constitutively active IL-18 receptor (Zip18R) (111), transgenic IL-12 (136), IL-15 (71, 137), locally released anti-CD73 antibody (103), and PD-1 knock-out (128). Overexpression of CCR2, CCR4, CXCR1/2, and CXCR4 reportedly improved CAR T cell trafficking to brain tumors in preclinical studies (118, 138–140).CAR T cell pretreatment with metformin/rapamycin also enhanced mitochondrial respiration, leading to improved CAR T cell efficacy in glioma (141).

C7R is particularly promising strategy, as a clinical trial has demonstrated that CAR T cells expressing C7R maintained stable blood levels from 3 hours to 4 weeks post-infusion unlike typical CAR T cells, whose blood levels decline rapidly (35). Co-expression of C7R in CAR T cells boosts STAT5 signaling, enhancing cell survival and proliferation during repeated antigen challenges while avoiding antigen-independent expansion (135). Preclinical models demonstrated that CAR T cells with C7R eradicate glioblastoma even at doses insufficient for efficacy in standard CAR T cells and exhibit increased resistance to apoptotic signals via BCL2 upregulation and FAS/CASP8 downregulation.

Zip18R, a synthetic IL-18-based receptor, improves CAR T cell expansion and cytokine production by activating MyD88-dependent signaling pathways (111). In glioma and sarcoma models, CAR T cells engineered with Zip18R displayed significantly greater antitumor efficacy than unmodified CAR T cells, with enhanced persistence and elevated secretion of effector cytokines such as IFN-γ, IL-2, and TNF-α. Importantly, Zip18R did not induce autonomous growth in the absence of antigen, supporting its use in solid tumors where chronic antigen exposure induces T cell dysfunction.

Similarly, intratumoral delivery of IL-12 has been shown to potentiate CAR T cell activity in preclinical glioblastoma models (136). A single local dose of IL-12 reshaped the tumor microenvironment by reducing regulatory T cells and inducing proinflammatory macrophage and CD4+ T cell infiltration. Combined with EGFRvIII-specific CAR T cells, IL-12 treatment led to durable tumor control and long-term survival, while avoiding systemic toxicity, demonstrating the power of localized cytokine modulation to rescue CAR T cell function in glioma.

Transgenic expression of IL-15 in CAR T cells targeting GD2 significantly enhanced tumor control in an orthotopic glioblastoma model (137). These IL-15-expressing CAR T cells showed improved persistence, greater tumor infiltration, and higher cytokine production, including IL-2, IFN-γ, and TNF-α, compared to conventional CAR T cells. The therapy yielded a complete response rate of 50% in preclinical studies, highlighting IL-15 as a potent enhancer of CAR T cell function and durability in the glioma setting.

Another promising strategy involves local release of an anti-CD73 antibody fragment by CAR-engineered NK cells (103). This approach enables tumor-specific suppression of adenosine-mediated immunosuppression by inhibiting CD73 activity in the glioblastoma microenvironment. In preclinical models, CD73-targeting constructs reduced adenosine accumulation, restored NKG2D expression on NK cells, enhanced cytotoxicity, and improved NK cell infiltration. These effects were associated with reorganization of the tumor immune landscape and suppression of glioma progression, establishing CD73 as a critical immunometabolic checkpoint in glioma therapy.

Checkpoint blockade via PD-1 knock-out is another method to restore CAR T cell function in glioblastoma (128). Using CRISPR-Cas9, PD-1 was deleted in EGFRvIII-specific CAR T cells alongside TCR and B2M to generate universal donor CAR T cells resistant to PD-L1-mediated immunosuppression. In preclinical glioma models, these triple-edited CAR T cells exhibited enhanced antitumor activity, elevated Th1 cytokine secretion (IFN-γ, TNF-α), and improved persistence following repeated antigen stimulation. *In vivo*, intraventricular administration of PD-1-deficient CAR T cells significantly prolonged survival and produced durable cures in some mice, demonstrating the importance of PD-1 pathway disruption in overcoming the glioma immune barrier.

CAR T cell trafficking to the brain tumor site can be enhanced through chemokine receptor engineering. CXCR1- and CXCR2-expressing CAR T cells exploited tumor-secreted IL-8 for selective homing (140). These modified cells showed superior intratumoral infiltration and persistence, which correlated with improved tumor clearance and induction of memory responses across preclinical models of glioblastoma and other solid tumors (91). CAR NK cells engineered with CXCR4 demonstrated chemotaxis toward CXCL12-producing gliomas, resulting in enhanced trafficking, tumor infiltration, and antitumor effects (139). CXCR4-overexpressing cells achieved complete remission in several treated animals and prolonged survival compared to controls, highlighting this receptor’s critical role in navigating the glioma chemokine landscape (111). Similarly, expression of CCR2 and CCR4 on CAR T cells could increase chemotaxis to CCL2/4/5/17/22-secreting gliomas (118).

Finally, metabolic reprogramming with metformin and rapamycin (Met+Rap) pretreatment significantly improved CAR T cell function under glioma-specific hypoxic conditions (141). This combination activated AMPK and suppressed mTOR signaling, inducing mitochondrial biogenesis via PGC-1α upregulation. As a result, CAR T cells exhibited enhanced oxidative metabolism, greater spare respiratory capacity, and resistance to exhaustion. *In vivo*, Met+Rap–pretreated CAR T cells achieved better tumor infiltration, persistent cytotoxicity, and extended survival in intracerebral glioma models.

Together, these multifaceted engineering strategies illustrate the growing sophistication of CAR T cell design, emphasizing the importance of combinatorial approaches to overcome the distinct immunological barriers posed by glioma.

## Conclusion

High-risk CNS tumors are resistant to standard treatments such as radiation and chemotherapy, and they remain the leading cause of mortality in pediatric oncology. As a result, there is a critical need for new therapeutic approaches to improve patients’ quality of life and achieve disease remission. Recent clinical studies of CAR T-cell therapy in CNS tumors have shown promising results, though there is still room for improvement.

A detailed analysis of these clinical studies has highlighted the initial antitumor activity of CAR T-cell therapy and its low toxicity. The presence of several clinically validated antigens for CNS tumor therapy opens opportunities for multi-target strategies to prevent antigen escape. Local delivery methods, such as intraventricular and intratumoral administration, show advantages over systemic delivery, ensuring higher concentrations of cells in the tumor while minimizing toxicity. Increasing the dosage and improving the persistence of CAR T-cells could also significantly enhance treatment efficacy. Therefore, with proper modifications, CAR T-cell therapy has the potential to substantially improve the survival rates of patients with high-risk CNS tumors.
