# Correction: CAR T cell therapy for central nervous system solid tumors: current progress and future directions

**DOI:** 10.3389/fimmu.2025.1689398

**Published:** 2025-09-23

**Authors:** Yaroslav Kaminskiy, Vitaly Degtyarev, Alexey Stepanov, Michael Maschan

**Affiliations:** ^1^ Department of Oncology and Pathology, Karolinska Institutet, SciLifeLab, Solna, Sweden; ^2^ Dmitry Rogachev National Medical Center of Pediatric Hematology, Oncology, and Immunology, Moscow, Russia; ^3^ Shemyakin-Ovchinnikov Institute of Bioorganic Chemistry, Russian Academy of Sciences, Moscow, Russia

**Keywords:** CNS tumors, glioma, DMG, DIPG, glioblastoma, CAR T, CAR T solid tumors, brain cancer

There was a mistake in Table 1 as published. “CAR T cells in blood; CD3+ T cells correlated with enhanced survival after therapy; ICV beneficial for multifocal tumors and IT beneficial for unifocal tumors; 75% transduction efficiency; IFN-γ, CXCL9 and CXCL10 increase in CNS; Best OS in dual IT/ICV delivery and CD62+ Tn/mem enrichment trial arm;”. The corrected text appears below.

“75% transduction efficiency; CAR T cells in blood; CD3+ T cells correlated with enhanced survival after therapy; ICV beneficial for multifocal tumors and IT beneficial for unifocal tumors; IFN-γ, CXCL9 and CXCL10 increase in CNS; Best OS in dual IT/ICV delivery and CD62+ Tn/mem enrichment trial arm”.

There was a mistake in Table 1 as published. “severe hypoxia at highest dose (without IL-2) likely due to congestion of pulmonary vasculature; 66% transduction efficiency”. The corrected text appears below.

“66% transduction efficiency; severe hypoxia at highest dose (without IL-2) likely due to congestion of pulmonary vasculature”.

There was a mistake in Table 1 as published. “antigen loss or decrease (potentially attributable to chemotherapy); 9/10 SD after 28 days; CAR T cell expansion; CAR T tumor infiltration; 20% transduction efficiency; IDO1, PD-L1, IL- 10 upregulation post therapy;”. The corrected text appears below.

“20% transduction efficiency; antigen loss or decrease (potentially attributable to chemotherapy); 9/10 SD after 28 days; CAR T cell expansion; CAR T tumor infiltration; IDO1, PD-L1, IL- 10 upregulation post therapy”

There was a mistake in Table 1 as published. “3/5 SD tumor necrosis; all (3) patients without C7R progressed after 1st infusion; longer PFS in C7R group; CAR T cell expansion and persistence in group with and without C7R; intratumor C7R transgene and residual GD2 detected in one patient analyzed; grade 4 CRS in one patient;”. The corrected text appears below.

“3/5 SD tumor necrosis; all patients without C7R progressed after 1st infusion; longer PFS in C7R group; CAR T cell expansion and persistence in group with and without C7R; intratumor C7R transgene and residual GD2 detected in one patient analyzed; grade 4 CRS in one patient”

There was a mistake in Table 1 as published. “no antigen loss; *in vivo* CAR T cell expansion; CAR T tumor infiltration in PD case after IV dose; regression of spinal cord tumor but not metastasis after IV dose; Tregs and MDSCs increase post therapy; dose limiting toxicity for IV but not ICV; manageable TIAN in all patients after ICV infusion correlated with CCL2 upregulation; PD associated with TGF-β upregulation; 57% transduction efficiency”. The corrected text appears below.

“57% transduction efficiency; no antigen loss; *in vivo* CAR T cell expansion; CAR T tumor infiltration in PD case after IV dose; regression of spinal cord tumor but not metastasis after IV dose; Tregs and MDSCs increase post therapy; dose limiting toxicity for IV but not ICV; manageable TIAN in all patients after ICV infusion correlated with CCL2 upregulation; PD associated with TGF-β upregulation”

There was a mistake in Table 1 as published. “8.1 vs 5.4 months PFS against chemotherapy alone;”. The corrected text appears below.

“8.1 vs 5.4 months PFS against chemotherapy alone”

There was a mistake in Table 1 as published. “17.5 vs 13.6 months OS against standard of therapy;”. The corrected text appears below.

“17.5 vs 13.6 months OS against standard of therapy”

There was a mistake in Table 1 as published. “IL-2-related CNS toxicity at 2.4 MIU dose; eosinophils infiltration;”. The corrected text appears below.

“IL-2-related CNS toxicity at 2.4 MIU dose; eosinophils infiltration”

The original version of this article has been updated.

**Table 1:** Cellular therapy clinical trials in CNS tumors.

Publication	Antigen	Cell Product	Conditions	Treatment	Injection Route	Injection Details	Additional therapy	Antitumor Activity	Interesting Observations
Brown et al. (2024) (15)	IL13Rα2	IL13Rα2-BBζ CAR T cells	rHGG (41 GBM, 6 AC, 2 DMG, 1 DAC, 7 grade 3 glioma)	2e6-2e8 cells (3-4 weekly doses)	IT + ICV	Rickham reservoirs (cells + 5 min saline flush)		2/58 CR; 2/58 PR; 29/58 SD	75% transduction efficiency; CAR T cells in blood; CD3+ T cells correlated with enhanced survival after therapy; ICV beneficial for multifocal tumors and IT beneficial for unifocal tumors; IFN-γ, CXCL9 and CXCL10 increase in CNS; Best OS in dual IT/ICV delivery and CD62+ Tn/mem enrichment trial arm
Bagley et al. (2024) (16); Bagley et al. (2025) (17)	IL13Rα2+EGFR	EGFR-IL13Rα2-BBζ bicistronic CAR T cells	18 multifocal rGBM (EGFR+)	1-2.5e7 cells (up to 2 doses)	ICV	Ommaya reservoir		8/13 tumor shrinkage; 1PR, 8SD	Pseudoprogression in 2 patients; CAR T cell expansion (less after second dose); CAR T cells in blood; grade 3 toxicity in 56% patients; manageable acute neurotoxicity 12-48h post-infusion (different from ICANS and TIAN); 2.5e7 cells max tolerated dose; IFN-γ, IL-2,IL-6, and TNF-α in CSF
Brown et al. (2022) (18)	IL13Rα2	IL13-zetakine+ CD8+ CTLs (GR KO, allogeneic)	6 rGBM (IL13Rα2+)	1e8 cells (4 doses over 2 weeks)	IT	Rickham reservoir (0.5 ml cells over 10 min + 1 ml PFNS flush over 2h)	IL-2 + dexamethasone	4/6 tumor necrosis	no antigen loss; potential allo-rejection
Brown et al. (2016) (19)	IL13Rα2	IL13Rα2-BBζ CAR T cells	1 multifocal rGBM (70% IL13Rα2+ tumor cells)	0.2-1e7 cells ( 6 IT + 10 ICV doses every 1-3 weeks)	IT+ICV	Rickham reservoir		1/1 CR (regression of 7 tumors)	IT injection prevented local but not distant tumor recurrence; predominantly CD4+ CAR T cells; no CAR T cells in blood; antigen loss leading to relapse
Brown et al. (2015) (20)	IL13Rα2	IL13-zetakine+ CD8+ CTLs	3 unifocal rGBM	1e8 cells (2 x 2-week cycles of 6 IT doses)	IT	Rickham reservoir (2 ml cells over 5-10 min + saline flush); externalized catheter (2 ml cells over 4h)		3/3 with antitumor signs (necrosis, antigen loss, no reccurence at resection border)	antigen loss
Gust et al. (2025) (21)	EGFRvIII + EGFR	EGFR-BBζ CAR T cells	3 rHGG, 1 AT/RT (over 10% EGFR+)	1-2.5e7 cells (5 – 10 weekly doses)	IT or ICV	Ommaya reservoir (5 ml cells over 5 min + saline flush)	–	1/4 SD	over 60% transduction efficiency; CAR T cells in blood but not in CSF, no CAR T cell infusion in over 50% of enrolled patients due to rapid disease progression
Choi et al. (2024) (22)	EGFRvIII + EGFR	EGFRvIII-BBζ CAR T cells (anti-EGFRwt TEAM secretion)	3 rGBM (EGFRvIII+ IDHwt)	1e7 cells (1-2 doses)	ICV	Ommaya reservoir (10 ml cells over 10 min)	–	3/3 tumor shrinkage	antigen loss; CAR T cells in blood
Bagley et al. (2024) (23)	EGFRvIII	EGFRvIII-BBζ CAR T cells	7 GBM (EGFRvIII+)	2e8 cells (up to 3 doses every 3 weeks)	IV	–	pembrolizumab	6/7 antigen decrease	21% transduction efficiency; no CAR T cell expansion; intratumor CAR T cells in one patient with earliest surgery (post 7 days) after infusion
Durgin et al. (2021) (24)	EGFRvIII	EGFRvIII-BBζ CAR T cells	1 rGBM (60% EGFRvIII+)	9.2e7 cells	IV	–	–	1/1 antigen decrease	antigen decrease; CAR T persistence fo 29 months; CD3 tumor infiltration post therapy
Goff et al. (2019) (25)	EGFRvIII	EGFRvIII-28BBζ CAR T cells	18 rGBM (EGFRvIII+)	1e7-6e10 cells (up to 10 doses)	IV	–	cyclophosphamide + fludarabine + IL-2	1/18 long term survival	66% transduction efficiency; severe hypoxia at highest dose (without IL-2) likely due to congestion of pulmonary vasculature
O'Rourke et al. (2017) (26)	EGFRvIII	EGFRvIII-BBζ CAR T cells	10 rGBM (6-90% EGFRvIII+)	1-5e8 cells	IV	–	–	1/10 SD; 2/5 antigen loss; 5/7 antigen decrease	20% transduction efficiency; antigen loss or decrease (potentially atributable to chemotherapy); 9/10 SD after 28 days; CAR T cell expansion; CAR T tumor infiltration; IDO1, PD-L1, IL-10 upregulation post therapy
Burger et al. (2023) (27)	HER2	HER2-28ζ CAR NK92 cells (irradiated)	9 rGBM (HER2+)	0.1-1e8 cells	IT	2 ml cells injected into resection cavity wall by 50-100 ul	–	5/9 SD	no antigen loss; PD all in lower dose group; 7 weeks PFS; 31 weeks OS
Vitanza et al. (2021) (28)	HER2	HER2-BBζ virus-specific T cells	1 AA and 2 ependymoma	1-2.5e7 cells (3 x 3-week cycles of 3 doses)	IT or ICV	CNS catheter	–	1/3 SD; 3/3 local CNS inflammation	CCL2 and CXCL10 increase in CSF and blood
Ahmed et al. (2017) (29)	HER2	HER2-28ζ virus-specific T cells	17 rGBM (HER2+)	1.7e6 - 1.7e8 cells (up to 6 doses every 6 -12 weeks)	IV	–	–	1/16 PR; 7/16 SD; 5/16 pseudoprogression	39% transduction efficiency; no CAR T cell expanion but 1 year persistence
Vitanza et al. (2023) (30); Vitanza et al. (2025) (31)	B7-H3	B7-H3-BBζ CAR T cells (DHFR mutein)	21 DIPG (H3K27M+)	0.1-1e8 cells (10-81 doses every 2 weeks)	ICV	Ommaya reservoir	–	1/18 PR; 15/18 SD; 8.6 months OS improvement	CAR T cells in CSF but not blood; CAR T cell enrichment due to methotrexate selection during manufacturing; CCL2, CXCL10, GM-CSF, IFN-γ, IL-15, IL-1α, IL-6, and TNFα upregulation in CSF
Tang et al. (2021) (32)	B7-H3	B7-H3-BBζ CAR T cells	1 rGBM (heterogeneous B7-H3 expression)	0.4-2e7 cells (6 weekly doses)	IT	Ommaya reservoir (1 ml cells over 10 min + 1 ml PFNS flush over 5 min)	–	1/1 tumor shrinkage	probable antigen loss; limited expansion in later infusion cycles
Tang et al. (2020) (33)	B7-H3	B7-H3-BBζ CAR T cells	1 anaplastic meningioma	0.2-1.5e7 cells (3 weekly doses)	IT	Ommaya reservoir (1 ml cells over 10 min + 1 ml PFNS flush over 5 min)	–	1/1 tumor stabilisation, necrosis and antigen decrease at infusion site	no trafficking to distant metastases; no CAR T cells in blood
Gu et al. (2025) (34)	GD2 + PSMA	GD2-28BBζ CAR T cells (iCASP9); PSMA-28BBζ CAR T cells (iCASP9)	5 rGBM, 1 rDMG	0.6-3.5e8 cells	IV	–	cyclophosphamide + fludarabine	4/6 tumor shrinkage; 3 CR; 3 SD	CAR T cell expansion but no correlation with response; CRS in 3 patients; no ICANS; low tumor burden in CR
Lin et al. (2024) (35)	GD2	GD2-BBζ CAR T cells (with or without C7R)	8 DMG, 2 medulloblastoma, 1 AT/RT	0.6-5e7 cells ( up to 4 doses every 6 weeks)	IV	–	cyclophosphamide + fludarabine + anakinra	2/11 PR; 5/11 SD; 9/10 improved neurologic deficit (3 weeks after infusion)	3/5 SD tumor necrosis; all (3) patients without C7R progressed after 1st infusion; longer PFS in C7R group; CAR T cell expansion and persistence in group with and without C7R; intratumor C7R transgene and residual GD2 detected in one patient analyzed; grade 4 CRS in one patient
Monje et al. (2024) (36); Ramakrishna et al. (2023) (37); Majzner et al. (2022) (8);	GD2	GD2-BBζ CAR T cells (iCssp9)	10 DIPG and 3 spinal DMG (H3K27M+)	1-5e7 cells (IV + 0-11 ICV monthly doses)	IV + ICV	Ommaya reservoir	cyclophosphamide + fludarabine (for IV only)	1/11 CR; 7/11 tumor shrinkage; 9/11 clinical improvement; 6.4 months OS improvement	57% transduction efficiency; no antigen loss; in vivo CAR T cell expansion; CAR T tumor infiltration in PD case after IV dose; regression of spinal cord tumor but not metastasis after IV dose; Tregs and MDSCs increase post therapy; dose limiting toxicity for IV but not ICV; manageable TIAN in all patients after ICV infusion correlated with CCL2 upregulation; PD associated with TGF-β upregulation
Liu et al. (2023) (38)	GD2	GD2-28BBζ CAR T cells (iCasp9)	8 rGBM (GD2+)	0.3–2e8 cells IV and 2.6-6.4e6 cells IT	IT + IV	Rickham reservoir	cyclophosphamide + fludarabine	4/8 PR; 1/8 SD; 1/8 pseudoprogression with antigen decrease	36% transduction efficiency; 7/8 patients with unmethylated MGMT; PD in (more advanced?) patients with IT infusion; CAR T cell expansion; T cell and macrophage infiltration after infusion
Qin et al. (2021) (39)	EphA2	EphA2-BBζ CAR T cells	3 rGBM (EphA2+)	6e7 cells	IV	–	cyclophosphamide + fludarabine	1/3 SD; 1/3 ealry tumor shrinkage	pulmonary edema likely due to lung EphA2 expression; CAR T cell expansion
Litten et al. (2023) (40)	MMP2	CLTX-28ζ CAR T cells	rGBM and AA (MMP2+)	0.8-1.5e8 cells (3 weekly doses)	IT + ICV	?		?	chlorotoxin (CLTX)-directed CAR T cells
Zhai et al. (2024) (41)	CD44 + CD133	CD44-CD133 CAR T cells ( IL7R; 4th generation)	4 rGBM	0.1-1e8 cells (up to 8 weekly doses)	IT?	Ommaya reservoir		tumor shrinkage	
Yao et al. (2023) (42)	–	TILs (anti-PD1 antibody secretion)	21 rGBM	monthly doses?	IV	–	?	1/7 PR and 2/7 SD in PD1-TIL group; 3/14 SD in TIL group	16.1 vs 11.2 months OS PD1-TIL against TIL; significantly improved OS in clinical responders (30.9 vs 10.7 months)
Quattrocchi et al. (1999) (43)	–	TILs	3 GBM, 3 (all recurrent)	0.3-1e9 cells (2 doses with 2 weeks in between)	IT	Ommaya reservoir (3-5 ml cells in saline)	IL-2	3/6 PR; 2/6 SD	
Kitahara et al. (1987) (44)	–	CTLs (autologous)	4 GBM, 1 AOD	5e7 cells (up to 13 doses twice per week)	IT	Ommaya reservoir (2 ml cells in HBSS)		2/5 PR and 50% tumor shrinkage	
Kruse et al. (1997) (45)	–	CD8+ T cells (alloreactive)	2 GBM, 2 AOD, 1 AA (all recurrent)	0.3-7.5e8 cells (up to 5 x 2-week cycles of 2-3 doses)	IT or ICV	Rickham reservoir (2-8 ml cells in HBSS with IL-2)	IL-2	3/5 SD	both GBM patients progressed and died (one from local and another from distant recurrence)
Ishikawa et al. (2004) (46)	–	NK cells	3 GBM, 4 AA, 1 AOD, 1 AOA (all recurrent)	0.2–3.7e9 cells IV and 0.4-2.3e9 cells IT (up to 3 doses over 9 months)	IT + IV		IL-2 + IFNb	3/9 PR 4/9 SD after 1 dose; 4/9 tumor shrinkage	
Lim et al. (2021) (47)	*-*	CIK cells	14 rGBM	2-6e9 cells (24 doses every 2 weeks)	IV	–	–	4/14 SD; 1/14 initial radiographic improvement	10 months PFS; 22.5 months OS; 5/14 patients survived over 2 years
Kong et al. (2017) (48)	–	CIK cells	91 GBM	0.1-2e10 cells (4 doses once a week, 4 doses every 2 weeks, 6 doses every 4 weeks)	IV	–	radiotherapy + temozolomide	2.7 months PFS improvement	8.1 vs 5.4 months PFS against chemotherapy alone
Dillman et al. (2009) (49)	–	LAK cells	33 GBM	1e9 cells	IT	clot placement	IL-2	OS improvement (20.5 months with a 1-year survival rate of 75%)	20.5 vs 15 months OS against standard of therapy; superior survival for patients received higher numbers of CD3+/CD16+/CD56+ cells
Dillman et al. (2004) (50)	–	LAK cells	40 r GBM	1-3e9 cells	IT	Ommaya reservoir or clot placement	IL-2	3.9 months OS improvement	17.5 vs 13.6 months OS against standard of therapy
Hayes et al. (2001) (51)	–	LAK cells	28 GBM or AA	0.5-5e9 cells (2 monthly doses)	IT	Ommaya reservoir (4 ml over 10 min)	IL-2	6.2 months OS improvement in grade 4 gliomas	eosinophilia correlated with survival
Sankhla et al. (1996) (52)	–	LAK cells	10 GBM or AA (all recurrent)	1-2.5e6 cells (up to 5 doses every 5 days)	IT or lumbar subarachnoid injection (for multifocal)		IL-2	2/10 PR	cystic transformation in responded patients
Hayes et al. (1995) (53)	–	LAK cells	12 GBM, 4 AA, 3 GS (all recurrent)	0.5-1e9 cells (up to 2 x 6-week cycles of 3 doses)	IT	5 ml cells	IL-2	2/19 CR; 4/19 PR	IL-2-related CNS toxicity at 2.4 MIU dose; eosinophils infiltration
Blancher et al. (1993) (54)	–	LAK cells	5 rGBM	3 doses every other day after surgery	IT	–	IL-2	no apparent benefit	IL-2 related toxicity
Jeffes et al. (1991) (55)	–	LAK cells	19 rGBM	2-58e9 cells (2 doses during surgery)	IT	–	IL-2 + phytohemagglutin	3/11 tumor shrinkage;	
Lillehei et al. (1991) (56)	–	LAK and ASL cells	9 GBM, 2 AA (all recurrent)	0.5-9e9 cells (two monthly doses)	IT	Rickham reservoir or clot placement (10-20 ml)	IL-2	no apparent benefit (10/11 died of progression)	9 months OS; local tumor growth and lack of lymphocytes after infusion in 3 evaluable patients;
Ingram et al. (1987) (57); Ingram et al. (1990) (58)	–	LAK cells	83 rMG	1e8-2e9 cells	IT	15-20 ml cells in plasma	76/83 initial response?; tumor shrinkage in several patients;	25/83 were living; 18/83 no evidence of recurrence;	
Merchant et al. (1988) (59)	–	LAK cells	13 rGBM	0.5-5e9 cells (2 doses with 1 - 2 weeks in between)	IT	Ommaya reservoir or multiple injections into surrounding tissue (3-5 ml cells in saline)	IL-2	5/13 SD, necrosis, lymphoid infiltration	
Jacobs et al. (1986) (60)	–	LAK cells	6 MG	5e7-1e10 cells	IT	№20 blunt brain cannula (5-15 ml cells in HBSS with multiple injections into surrounding tissue)		no apparent benefit	

